# Challenges and Vision for Standardization of Biopolymer Data Sets for Machine Learning

**DOI:** 10.1021/acs.biomac.6c00211

**Published:** 2026-06-10

**Authors:** Jessica N. Lalonde, Defne Circi, Babetta L. Marrone, Stefan Zauscher, L. Catherine Brinson

**Affiliations:** † Bioscience Division, 5112 Los Alamos National Laboratory, P.O. Box 1663, Los Alamos, New Mexico 87545, United States; ‡ Department of Mechanical Engineering and Materials Science, 3065 Duke University, 144 Hudson Hall, Campus Box 90300, Durham, North Carolina 27708, United States

## Abstract

Machine learning (ML) is transforming materials research, yet potential for biopolymer discovery remains constrained by fragmented data and nonstandardized reporting. Biopolymers differ significantly from synthetic polymers, requiring specialized approaches to represent their biosynthetic origins, hierarchical structures, and application-specific metrics. In this Perspective, we identify three core challenges limiting biopolymer representation: information encoding, data quality, and data sharing. We describe the most pressing issues and propose commensurate approaches to address each key challenge. Recommendations include the design and adoption of biopolymer-specific fingerprinting and representation frameworks, development of hybrid human-large language model (LLM) data extraction strategies, and expanding Findable, Accessible, Interoperable, Reusable (FAIR)-compliant repositories. We propose a robust foundation to define interoperable, high-quality data sets that capture the full context of biopolymer materials. Standardized metadata, shared ontologies, and community-driven infrastructure would enable scalable, reproducible workflows and accelerate the ML-driven development of biopolymers.

## Graphical abstract

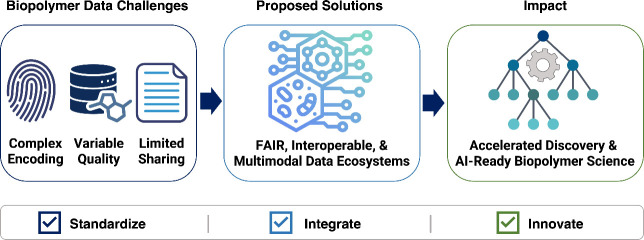

## Introduction

Machine learning (ML) and artificial intelligence (AI) are transforming materials science by enabling data-driven discovery, optimization, and property prediction^1–6^ across diverse materials systems, including semiconductors,^7,8^ biomedical materials, and polymers.^9–13^ The acceleration of polymer development through ML stems from uncovering structure–property relationships, automating feature extraction, and facilitating high-throughput screening,^14–17^ thereby increasing discovery and development potential and experimental efficiency beyond traditional approaches.^18–23^ Integrating ML with computational simulations and experimental data sets has driven significant advances in polymer engineering.^5,24,25^ Data science-supported development of polymers has been recently driven by high-quality data management toolkits,^26–29^ improved computational resources,^17^ and extensive open-access data repositories such as Polymer Genome,^30^ MaterialsMine,^31,32^ the CRIPT framework,^33^ and PolyInfo.^34^

While ML for synthetic polymers has grown rapidly, its application to biopolymers remains comparatively limited.^35,36^ Here, we define biopolymers as polymers derived from biological sources, or produced by microbes in a biosynthesis process.^37,38^ Biopolymers are a chemically diverse group of biomolecules consisting of, for example: biopolyesters with linear chain backbones and short chain branching, such as polyhydroxyalkanoates (PHAs); linear or branched polysaccharide chains comprised of sugar units; and proteins with amino acid residues and complex hierarchical structures. Biopolymers show significant promise as alternatives to conventional plastics in many material applications, including for sustainable food packaging, and in biomedical applications ranging from medical devices to tissue engineering scaffolds.^3,39–43^ However, their unique structural diversity, biosynthetic variability, and lack of well-defined structure-sequence mappings introduce variability not typically encountered in synthetic polymers.^23,44,45^

Several factors make it challenging to construct uniform, interoperable data sets suitable for ML-driven discovery for biopolymers. Data sets on key material characteristics such as mechanical properties, gas permeability, thermal stability, biocompatibility, and environmental stability are often scattered throughout unstructured data sets across the literature.^37,46^ Furthermore, this data also commonly lacks uniformity in measurement protocols, metadata, and semantics.^35,44,47–50^ While text-mining, information encoding, natural language processing (NLP), and large language model (LLM) methods have been employed for data extraction in conventional polymer research,^18,51^ their application specifically to biopolymer data sets remains limited due to nonstandardized terminology and featurization.^14,18,52–59^ This lack of standardization impedes efforts to systematically organize both historical and current experimental data.^13,18,58,60^

As biopolymer production grows, data standardization will be essential for ensuring interoperability and usability across independently generated data sets for building more robust ML workflows.^49,61–65^ Enhancing data standardization through structured metadata, NLP-based extraction tools, and interdisciplinary collaboration would improve predictive accuracy, facilitate data sharing, and accelerate the discovery and development of sustainable polymer alternatives to conventional polymers. In this perspective, we examine key challenges in biopolymer data set assembly and offer recommendations and resources for standardization (Figure 1). By suggesting standardized data set practices and framing biopolymers within the materials data space, we aim to foster discussion, communication, and collaboration across the broader biopolymer research community to improve ML-driven research efficiency, which ultimately advances biopolymer innovation and usage.

**Figure 1:**
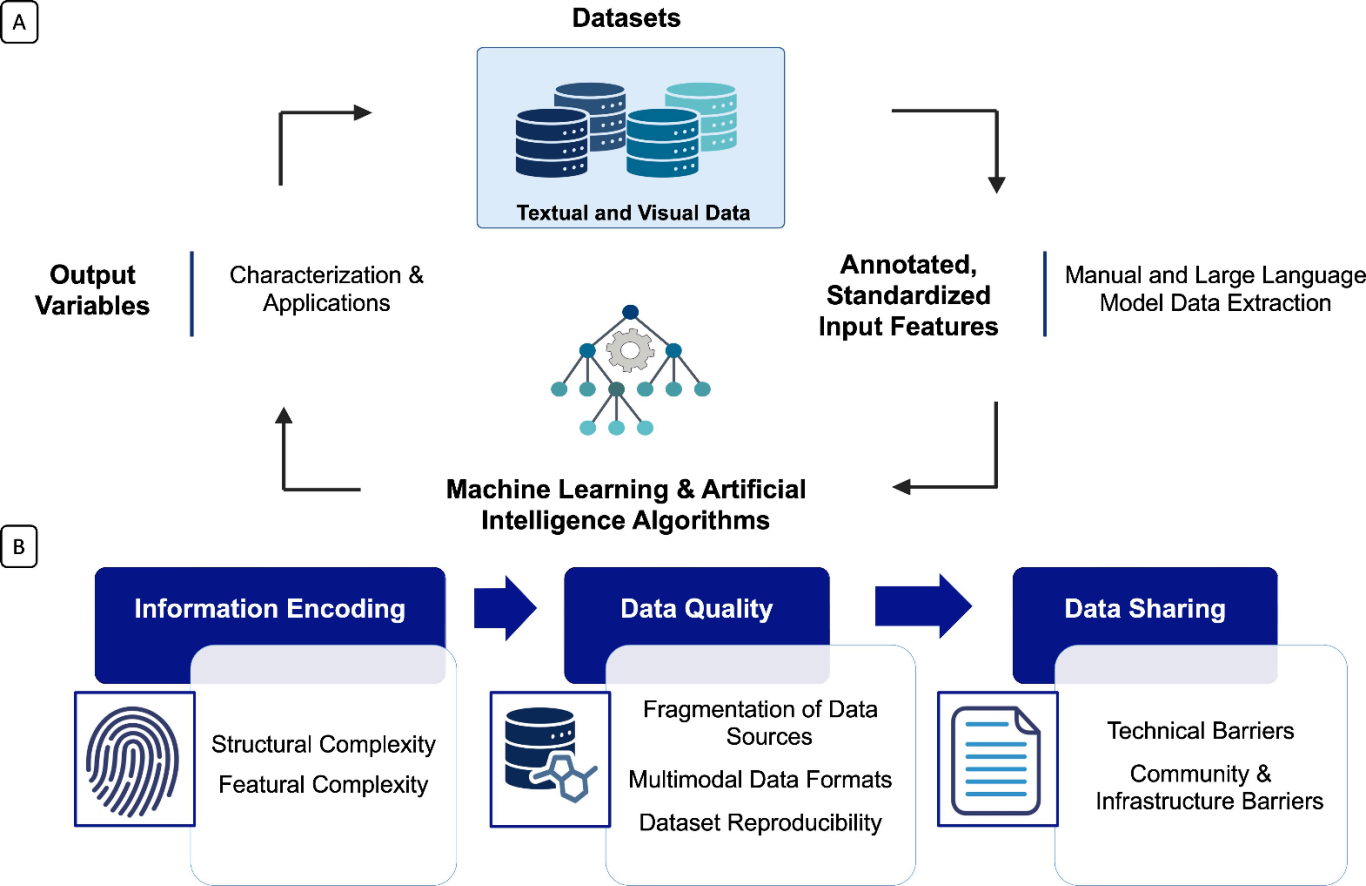
Challenges and opportunities in machine learning (ML)-driven biopolymer data workflows. (A) Here we depict an idealized ML and AI workflow for biopolymer materials, in which both textual and visual data from the literature or data repositories are annotated and aggregated into structured data sets. These data sets undergo standardized preprocessing and information encoding to serve as input features for ML/AI models that generate predictions, classifications, and property evaluations. The resulting outputs can then be standardized and reintegrated into future data sets, forming a cyclical data flow that promotes continuous improvement in data quality and usability. (B) We describe barriers to realizing this idealized workflow focused on three key challenges specific to biopolymers: information encoding, data quality, and data sharing.

## 1 Information Encoding

Biopolymers pose encoding challenges that overlap substantially with those encountered in synthetic polymer systems, including hierarchical conformations, branching and cross-linking distributions, and environmentally responsive behavior.^19,66–68^ These challenges are well-investigated within the synthetic polymer community. However, biopolymers often combine these phenomena with biosynthetic variability, heterogeneous feedstocks, and multiscale biological organization, creating additional challenges for standardized representation and machine-readable encoding. Consequently, many features relevant to biopolymer performance, such as conformational ensembles, hydration-dependent states, degradation pathways, and processing-history effects, are only partially captured by existing molecular representations and property schemas.^69–77^ Inconsistent reporting of biosynthesis, processing history, testing conditions, and environmental exposure further complicates integration of data sets across studies.

Scheme 1 summarizes two information encoding challenge classes, structural and featural, and maps them to specific recommendations. At a high level, we propose the following: expand existing structural encodings to include tailored extensions specific to biopolymers; standardize processing, and application-specific metadata, and utilize existing tabular data for these purposes; and apply node-based frameworks plus NLP and self-supervised learning (SSL) pipelines to generate uniform, machine-readable features. Together, these steps normalize the feature space across diverse sources so models can link structure, context, and measured outcomes more reliably. The following subsections elaborate the challenges and the corresponding recommendations listed in Scheme 1.

**Scheme 1:**
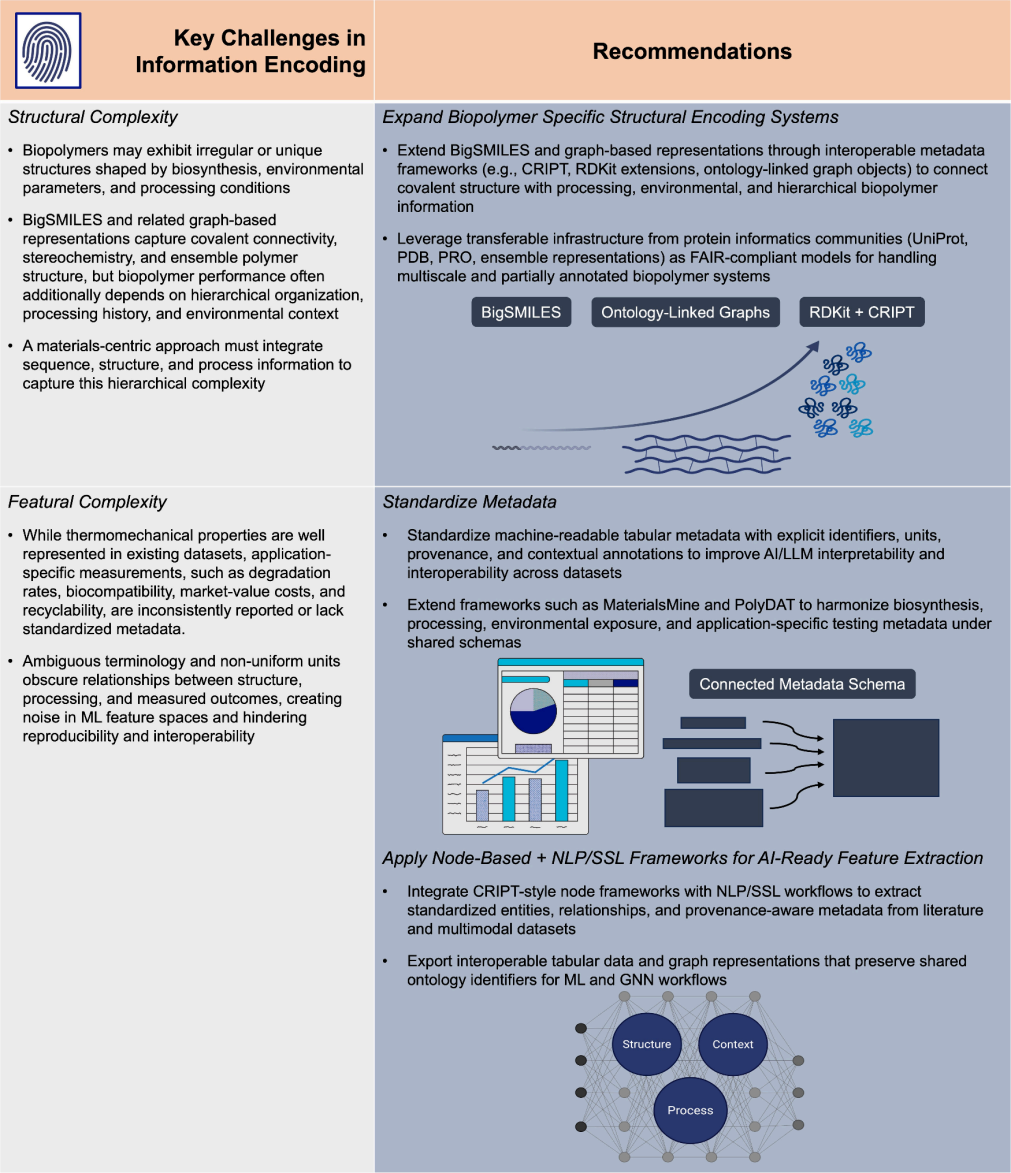
Roadmap for Improving Information Encoding of Biopolymer Datasets^a^

a Here we describe key challenge areas for future work and corresponding suggestions for improving information encoding in ML-driven biopolymer research. On the left, we identify two encoding challenges: structural complexity and context-dependent featural complexity. On the right, we present three recommendation areas to address these challenges through standardized representations, metadata schemes, and applying node-based frameworks for feature extraction.

### 1.1 Key Challenges in Information Encoding

#### 1.1.1 Structural Complexity: Representing Biopolymer Diversity

Biopolymer structural encoding is hindered by organism-dependent biosynthesis and environmental sensitivity, which produce batch-to-batch variability in monomer distribution, molecular-weight and dispersity profiles, stereochemistry/enantiomeric ratios, branching, crystallinity, and conformational states.^78–82^ Processing steps such as solvent casting, extrusion, or compression molding further change morphology and properties.^83–85^ Recent work by Clarke et al.^86^ further demonstrates that for PHAs, processing conditions such as thermal history, shear, and solvent exposure can measurably alter stereoregularity and tacticity distributions, with downstream consequences for crystallization behavior, mechanical performance, and degradation profiles. These findings highlight the need for encoding schemes that explicitly capture processing-dependent structural evolution rather than nominal polymer identity alone. Beyond primary composition, many macromolecular biopolymers adopt higher-order conformations via hydrogen-bonding, hydrophobic interactions, and other noncovalent effects.^82,83,87^ Examples include spider-silk fibroin with crystalline β-sheet domains interspersed with amorphous segments;^83,88,89^ and cellulose, starch, and chitin derivatives with characteristic chain packing and microfibrillar order.^90–93^ used alone or as additives or fillers in biomaterial formulations.

Structural information is currently under-represented in biopolymer encoding systems. Most existing encoding systems, such as RDKit,^26^ the Simplified Molecular Input Line Entry System (SMILES/PSMILES),^28,29,94^ and similar linear notations – while extensive and well-documented – are optimized for small molecules with regular repeating connectivity.^95,96^ These systems have been then been adapted to work for a small class of synthetic polymers.

BigSMILES strings are chemically detailed line notations which build on SMILES and provide chemically rigorous and extensible encodings for molecular and polymer structure, including stereochemistry, branching, sequence distributions, and stochastic ensemble connectivity.^97^ BigSMILES enables explicit representation of distributions of covalently bonded entities and supports tagging of structural features that may be linked to experimentally measured attributes through interoperable frameworks such as CRIPT. However, many biopolymer systems exhibit additional hierarchical, environmentally responsive, and processing-dependent behavior that extends beyond covalent structure alone.^14,36,85,98^ As a result, conformational heterogeneity, mesoscale organization, hydration state, degradation environment, and processing history often require linked metadata, ontology-aware annotations, and multimodal contextual representations beyond line-notation chemistry encodings alone. In this way, BigSMILES effectively captures covalent ensemble structure, and can be linked to this extended information through interoperable metadata systems.

However, even when biopolymer crystalline structure or monomer identity is well documented, as is the case with polyhydroxyalkanoates (PHAs) and polylactic acid (PLA),^87,99^ structural descriptors such as morphology, chain conformation, and hierarchical ordering are often missing or scattered across supplementary files and figures. In contrast, material properties such as tensile strength or elastic modulus are typically tabulated but decoupled from their structural context, forcing researchers to extract and realign this information manually for ML use.^20,22,100^ This separation between structural detail and property measurement continues to constrain the creation of interoperable, high-fidelity biopolymer data sets.

Proteins represent a unique subclass of biopolymers whose detailed structural information is captured in repositories such as the Worldwide Protein Data Bank (PDB),^101^ the Protein Ontology (PRO),^102^ and the Universal Protein Knowledgebase (UniProt),^103^ and the Proteins*Plus* interactive tool^104^ for protein–ligand complex mining and modeling. However, while these repositories - and the extensive body of work associated with them - provide mature examples of protein representation, these resources were not developed for materials-specific applications and do not connect sequence and conformation data to materials properties information or processing history. Specifically, while protein sequence–structure–function relationships are exceptionally well characterized within these repositories and other methods,^105^ protein data sets are not typically organized around materials performance metrics; comprehensive, standardized data linking proteins to mechanical, thermal, degradation, and processing-dependent properties remain limited. This gap parallels challenges across the broader biopolymer community, where materials-relevant performance data are fragmented or inconsistently reported despite advances in structural characterization.

Recent AI-based protein structure prediction tools such as AlphaFold,^106^ RoseTTAFold,^107^ and the LLM-based model ESM-2 ^108^ have enabled great strides in predicting macromolecular structure from sequence. These frameworks have provided examples of how self-supervised learning embeddings can capture structure–function relationships from sequence alone. Consequently, powerful tools such as graph-based structure encodings and learned contact maps are now common in these communities. Yet, these models are limited in their ability to capture environmental factors and molecular interactions that affect structure, conformation, and ultimately, function in a particular context.^109^ Bridging this gap in regard to protein biopolymers as biomaterials requires a materials-centric representation that unites atomistic, mesoscopic, and process-level descriptors with experimentally measured outcomes.

#### 1.1.2 Featural Complexity: From Processing Conditions to Application Metrics

Thermomechanical properties, for example, glass transition temperature,^100,110–113^ melting temperature,^19,114^ elastic modulus^115–117^ are generally well covered in existing biopolymer data repositories and are widely ML-amenable.^14,20^ In contrast, other features representing processing conditions and application-specific measurements suffer from inconsistent reporting in the literature and a lack of metadata schemas in repositories.^16,20,67,118–120^ Ambiguity in terminology and nonuniform units/labels further obscure comparisons and complicate aggregation.^121–123^ These inconsistencies reduce searchability, impede semantic alignment, and directly degrade NLP-based data extraction. LLMs can often extract structured information from tabular data sets with reasonable accuracy when tables preserve implicit conditions, explicit formatting, and row–column relationships.^18,51,124,125^ However, important experimental assumptions, inherited conditions, provenance information, and hierarchical relationships may still remain implicit even within tables, particularly in materials science data sets containing merged cells, abbreviations, unconventional formatting, or context distributed across multiple rows and captions.^124^ Circi et al. (2024) further demonstrated that LLMs can successfully recover composition and property information from polymer composite tables while still struggling with ambiguously inherited conditions and nonstandard table layouts that obscure contextual interpretation.^125^ The net effect of these challenges is a constrained, noisy feature space that weakens the ability of ML to capture structure–processing–property linkages for biopolymers.

### 1.2 Recommendations for Standardizing Information Encoding

To address the challenges outlined above, we propose a multifaceted strategy to improve information encoding of biopolymers. These recommendations draw on recent advances in cheminformatics and ML to move toward Findable, Accessible, Interoperable, and Reusable (FAIR)-compliant data sets which are inclusive of application-specific metrics.^126–129^

#### 1.2.1 Expand Biopolymer Specific Structural Encoding Systems

We propose using graph-based encodings and modular extensions that preserve hierarchy while remaining ML-ready. Recent frameworks demonstrate how to implement this strategy and move beyond the previously described limitations:

- **Leverage graph representations and multitier descriptors**: Antoniuk et al.^130^ introduced a periodic polymer graph representation that captures the periodicity of polymers and generates descriptors without manual featurization. Similarly, PolyMetriX^131^ encodes multiscale descriptor frameworks, from monomer identity to supramolecular assembly, while preserving interpretability.^132,133^

- **Expand with BigSMILES**: Building on these advances, we propose the community expand on the use of “BigSMILES” to incorporate stereochemical variability including enantiomers, branching/cross-link density, molecular-weight distribution, sequence/block information, and flags for environment-responsive states. This augments connectivity with minimal, standardized hierarchical tags.
- **Expand RDKit and graph modules**: Expanding RDKit with biopolymer-focused modules would enable integration of biosynthesis, processing, and environmental descriptors to molecular graphs. Paired with graph-based encoding frameworks, such as GraphIE reported by Qian et al.^134^ where nodes represent residues, additives, or functional motifs and edges capture spatial and reactive relationships, and graph neural networks (GNNs),^135,136^ these systems could then learn cross-scale correlations and represent biopolymer complexity in a consistent and flexible way.^14,135^ This process has recently been demonstrated with PolymerGNN, a multitask ML architecture developed by Queen et al.^137^ for predicting synthetic polymer properties based on polyesters with multiple levels of structural complexity such as branching and copolymerization.
- **Bridge to existing protein resources**: Leveraging existing FAIR-compliant infrastructure centered around proteins, like the UniProt, PRO, and PDB resources, that already standardize sequence–structure relationships would enable linking of sequence to conformation and materials-level descriptors for protein-based biopolymers. These resources provide excellent models for metadata-rich, interoperable repositories. Using the Chemical Entities of Biological Interest (ChEBI)^138^ database would further anchor a controlled vocabulary for monomers/functional groups. These chemical and protein-focused precedents are directly relevant for the biopolymer representations and other data encoding pipelines that we discuss throughout this perspective. For instance, even the intrinsically disordered regions of biopolymers present similar encoding assumptions as protein-based biopolymers,^105^ and the protein informatics community has developed specialized infrastructure for handling edge cases within these systems. Piovesan et al. (2025) describe how MobiDB integrates disorder predictors, ensemble-property annotations, and probabilistic functional mappings from experimental evidence to represent intrinsically disordered proteins within FAIR-compliant repositories.^139^ MobiDB provides an excellent example of addressing fuzzy functional annotations and disorder predictors within FAIR infrastructure. Kannan and Naganathan (2025) further advocate for a “sequence–ensemble–function” framework in which proteins are treated as dynamic conformational ensembles rather than single static structures, providing a best-practices example of ensemble representations.^140^ Similarly, Von Bülow et al. (2025) review ML-based approaches that combine protein embeddings, ensemble modeling, and probabilistic sequence–ensemble relationships to handle conformational heterogeneity and incomplete annotations in intrinsically disordered systems.^105^ These best practices on handling edge cases from the protein informatics community can be further extended into biopolymer ontologies.

Building on these recommendations, we propose the following action items:

1. Extend materials library mapping with BigSMILES. Publish a minimal specification with examples of commonly referenced biopolymers, such as standard compositions of PLA, polyhydroxybutyrate (PHB) and other common PHAs, and cellulose acetate.
2. Provide open reference implementations (RDKit extensions + converters to graph objects) so data sets can export unified structural representations.
3. Launch a structural ontology that links to, and does not duplicate, PRO, PDB, UniProt, ChEBI, and digitized resources such as the Polymer Handbook,^141^ which includes comprehensive polymer physical property data. Such digital resources could be further curated as an ontology foundation for biopolymers with machine-readable fields for stereochemistry, molecular-weight distributions, crystallinity, branching, hierarchical motifs, and physical material property data. Within polymer informatics, an ontology is defined as a structured semantic framework that formally defines entities, properties, relationships, and controlled vocabulary associated with a material system, enabling interoperable and machine-readable organization of data across experiments and repositories. Unlike molecular representations, which describe or featurize individual materials, ontologies define how information is annotated, connected, and interpreted across data sets.^138,142^ In biopolymer systems, ontologies may therefore link sequence information, processing history, morphology, environmental conditions, and measured properties within a shared and reusable metadata framework.

A unified toolkit of this kind would expand polymer informatics beyond linear synthetic systems to encompass the wide range of biologically derived materials, enabling ML models to capture realistic structural variability within a shared ontological framework.

#### 1.2.2 Standardize Metadata

Herein, we refer to metadata as the structured information that describes the context, content, and provenance of a data set, which makes the primary data FAIR-compliant. Metadata are descriptive labels and attributes that explain how, when, and under what conditions data were generated, measured, or manipulated, and may be distinct for environmental, processing, and other contexts. Compared with structural and basic property fields,^14,29,30,143^ metadata related to biosynthesis, extraction, processing, and application environments of biopolymers are the least standardized and most fragmented, yet they have a profound influence on measurable outcomes.^3,40,144–146^ To capture these descriptors we propose creating a shared schema that captures metadata with clear units, controlled vocabularies, and links to established standards.^141–143^ Several existing resources provide actionable examples on how to implement this strategy:

- **Model after MaterialsMine**. As illustrated in Figure 2, existing node/attribute schemas such as MaterialsMine show how solvents, heating, mixing, extrusion, and characterization can be encoded as graph relationships with consistent tags.^31,147^ The MaterialsMine framework also defines consistent tags and descriptors, as summarized recently in a compilation of materials domain-level ontologies.^148^ We propose extending this approach to biopolymer-specific contexts.
- **Define the scope of the schema**. For instance, a MaterialsMine extension for PHB may include biosynthesis conditions, extraction conditions, processing conditions, and application-specific environmental responses.
- **Implement a “digital twin” capable of using physical standards**. Develop machine-readable representations of standardized testing protocols (such as ASTM D6400, ASTM D5338–15–2021, ISO 17088:2021)^149,150^ by encoding experimental conditions, units, permissible ranges, and reporting constraints into interoperable metadata schemas that can support future digital twin models of biopolymer processing, degradation, and performance.^151,152^

**Figure 2:**
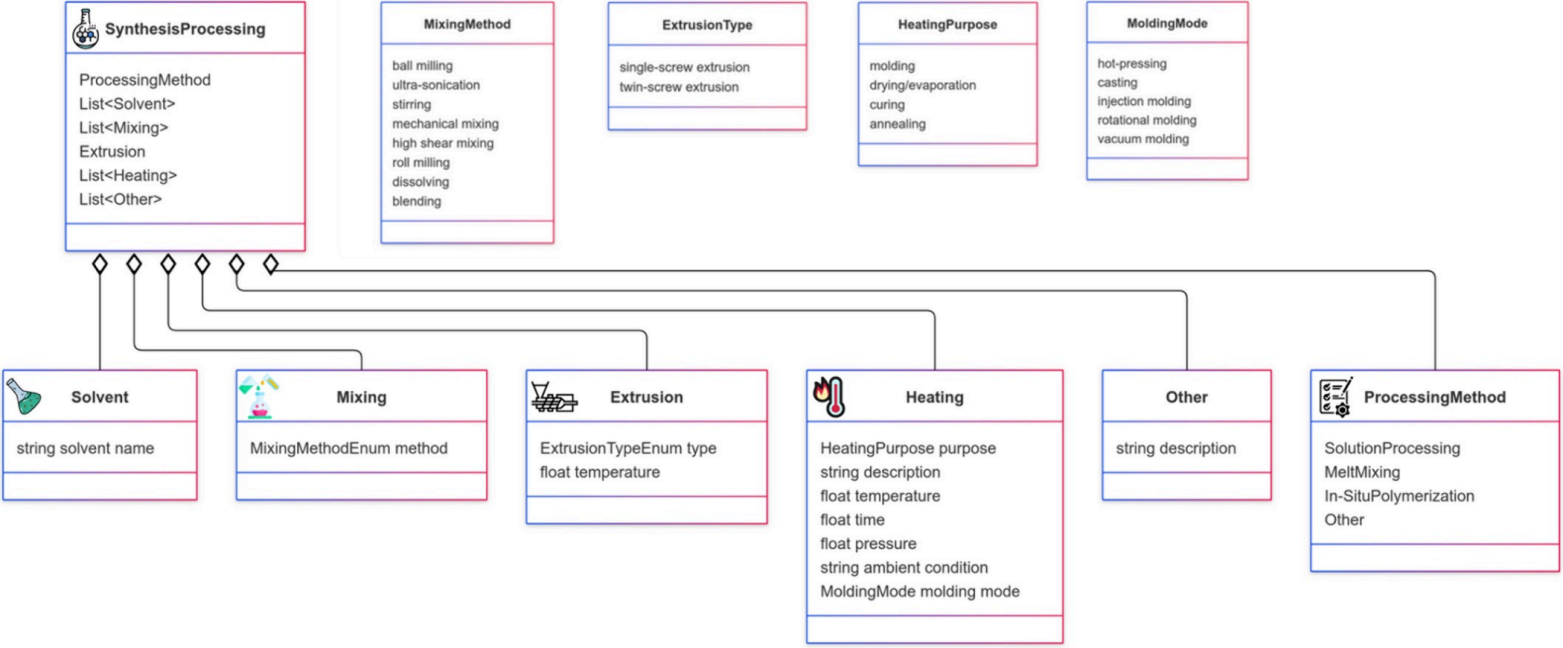
Polymer synthesis and processing scheme visualization with MaterialsMine. Here we present snapshots from a structured representation of synthesis and processing metadata (solvents, heating, mixing, extrusion) enabling standardized ML inputs and interoperability, which can be adapted for biopolymers. Adapted from MaterialsMine and created by Erdantic v1.1.0.post1 (https://github.com/drivendataorg/erdantic).^147^

Based on these examples, we propose the following action items:

1. Build a biopolymer processing, environmental response, and application-specific metadata library. This library would be analogous to the MaterialsMine framework, or perhaps a more specific application of PolyDAT^153^ in which every characterization on a relevant species is annotated, but tailored to biological/environmental interactions. Additionally, publish JSON/YAML schemas and controlled vocabularies. These items complement Section 1.1.1.4’s *structural ontology*. Data sets should ideally reference both.
2. Provide standard extract/validate tooling that maps literature terms to the vocabulary. For example, mapping “industrially compostable” to specific temperature/humidity/time profiles; and exports a useable format. As MaterialsMine does not currently have these entities, expanding the capabilities of existing resources would be useful.
3. Encode the standards above as a digital-twin schema to enable consistent collection, training, and cross-study validation; and align with FAIR standards.

#### 1.2.3 Apply Node-Based + NLP/SSL Frameworks for Expanded AI-Ready Feature Extraction

We make a distinction here between a ‘fingerprint’ and a ‘representation’ for biopolymers. A fingerprint is a machine-readable vector, or graph-embedded feature set, which summarizes a sample’s identity, structure, and context. These values are typically lossy when used for AI, meaning the specific, subtle patterns or ‘signatures’ of the feature space may be left out by a generative model during its training or during the output generation process. For instance, a SMILE string for a polymer may be converted to a Morgan fingerprint (a molecular representation used widely in structure–activity (QSAR) studies to encode local atomic substructures as a fixed-length vector). This contrasts with a ‘representation’, which captures the chemistry of a molecule with as much fidelity as possible, such as a SMILES or BigSMILES string. For example, a PLA film’s representation might link to additional libraries which can expand beyond the chemistry of a molecule to include monomer/ratio, molecular weight, stereochemistry, branching/cross-link descriptors, crystallinity, processing history, and application environments. The fingerprint data could then be paired with measured outcomes paired with the representation within the greater context of the information encoded. Each element would be standardized with controlled ontology terms so that fingerprints and representations from different sources are interoperable.^134–137^ As ML and AI models improve, future work should aim to decouple these concepts and distinguish between capturing a molecule, the state of a molecule, and the overall representation of a biopolymer compared to its fingerprint.

While MaterialsMine and similar infrastructures provide robust, standardized metadata libraries for capturing processing parameters, the Community Resource for Innovation in Polymer Technology (CRIPT) data model developed by Walsh et al.^33^ offers a node-based, FAIR-compliant system for linking those metadata to molecular and computational representations, which also includes a controlled vocabulary. Together, these systems enable complete traceability from raw synthesis to measured property data. For instance, MaterialsMine extensions supply the controlled vocabularies and descriptors that define each process or condition; whereas CRIPT organizes these relationships into interoperable graph nodes connecting materials, processes, computations, and data. This integration ensures that encoded biopolymer records carry both contextual and structural meaning, improving interoperability across repositories and enhancing ML model performance.

Therefore, we recommend using complementary frameworks to construct representations of the biopolymer data:

- **CRIPT as the graph node backbone**. Polymer data in the CRIPT data model is encoded within a FAIR-driven ecosystem.^127,129^ CRIPT organizes polymer data as interconnected nodes; describing material identity, processing history, computations, and resulting experimental or simulation-derived data that collectively define relationships between structure, processing, and measured properties. CRIPT also explicitly incorporates controlled vocabularies and federated storage architectures, allowing experimental and computational data sets to remain distributed across repositories while being linked through a unified metadata and identifier framework.^33^ Fundamentally, CRIPT organizes materials as nodes for *material*, *process*, *computation*, and *data*, and links BigSMILES to these nodes.^97^ The use of BigSMILES within frameworks such as CRIPT further emphasizes experimentally measured quantities and provenance-aware annotations rather than inferred structural assumptions, enabling polymer data sets to evolve as characterization methods improve. We therefore recommend extending existing CRIPT-controlled vocabularies with BigSMILES and extend node attributes to better accommodate biopolymer-specific processing, environmental, and application-dependent metadata using interoperable biopolymer data sets. The existing MaterialsMine workflow for processing metadata is shown in the example in Figure 2. We note that MaterialsMine does not currently include any biopolymer data, but rather it is the workflow and structure of the system which can be translated biopolymer-specific data:
  - **New node types/attributes**: From synthesis processing, mixing method, extrusion type, heating purpose, and molding mode nodes as listed in Figure 2 as examples, we can extend CRIPT node structures with additional biopolymer-specific attributes and controlled vocabulary terms describing fields such as biosynthesis conditions, degradation environment, biocompatibility assays, barrier testing, hydration state, or microbial exposure. For example, synthesis-processing nodes may include metadata describing metabolic pathway, fermentation conditions, compost temperature and exposure duration, assay protocol information, or gas permeability testing conditions.
  - **Mappings**: Each new metadata field maps to controlled vocabulary terms, ontology identifiers, and digital-twin-compatible standards so that CRIPT graphs, metadata libraries, and downstream ML workflows remain interoperable and synchronized.
  - **Structural slots**: Pointers to BigSMILES/RDKit graph objects (Section 1.1.1.4) should be included so a CRIPT record holds both structure and context within a unified representation framework.
- **NLP + SSL for labeling**. Integrate LLM-based annotation with SSL to mine unlabeled literature and automatically populate CRIPT nodes with standardized terms and units.^154^ For instance, Gao et al.^155^ have recently demonstrated success with GNNs and SSL for predicting polymer properties, which may be further extended to predicting application-specific outcomes given appropriate encoding libraries. However, automated extraction workflows will still require human oversight, particularly for ambiguous terminology, inherited experimental conditions, and multimodal contextual relationships, which has been demonstrated in other fields such as in the analysis of chemical reaction networks.^156^ Accordingly, future development of operator-focused validation software that enables rapid and intuitive review, correction, and provenance-aware verification of NLP- and SSL-generated annotations by a human domain expert could substantially improve data set reliability while reducing the burden of manual curation. We recommend the development of such a software package to streamline this process.
- **Resulting representation exports**. From CRIPT graphs, export standardized, tabular, feature vectors for ML and graph objects for GNNs. Both data sources should reference the same ontology IDs to ensure data set interoperability.^157–159^

Based on these recommendations we propose the following action items:

1. Develop an NLP pipeline that detects entities/values in text/tables/figure captions (“ASTM D5338”, “55 °C”, “proteinase K, 2 U/mL”) and normalizes units via the metadata library.
2. Train SSL/GNN models to learn embeddings, by which we mean vectorized, numerical representations of structural and contextual information such as molecular graphs, processing conditions, or environmental parameters, that capture relationships between similar samples.^38,46^ These embeddings allow the model to recognize patterns and semantic similarity even when explicit metadata are missing, improving extraction recall by identifying related entities or conditions across partially labeled data sets and making automated data extraction more complete and more accurate.
3. Implement a human-in-the-loop pass to validate uncertain fields. Incorporate explicit notes to separate limitations and highly uncertain values, such as ambiguous labels in visual data. These fields should then be further annotated with a hybrid process incorporating a domain expert.

With node-based encodings tied to a shared vocabulary and automated curation process, biopolymer data sets become findable and mergeable across laboratories and repositories, and ML models can incorporate realistic structural variability, and processing and application contexts. This not only improves human interpretability but also enhances the performance of ML models by reducing noise in training data.

## 2 Data Quality

Building on Section 1.1’s focus on information encoding, we next address data quality, the degree to which encoded information is complete, consistent, and reproducible enough to support ML.^126,160^ Specifically, we describe several reasons why existing biopolymer data are difficult to reuse or reliably train ML and AI models. Scheme 2 summarizes this section’s challenges and recommendations addressing data quality. In biopolymer data, the dominant obstacle is fragmentation, which we define here as relevant information being dispersed across many sources such as literature text, figures, supplements, lab notebooks, and simulations.^128,161^ Biopolymer data sets also appear in multimodal formats: textual, tabulated, and visual formats, each requiring distinct extraction and encoding pipelines.^79,162^ Finally, data set reproducibility depends on transparent preprocessing, versioning, and standardized identifiers so that independently compiled data sets can be merged or reanalyzed without ambiguity.^125^ Multiple sources can be compiled into a single data set, but only after normalization or alignment of metadata, units, scales, and feature definitions so records are directly comparable for ML.^78,79,162–164^

**Scheme 2:**
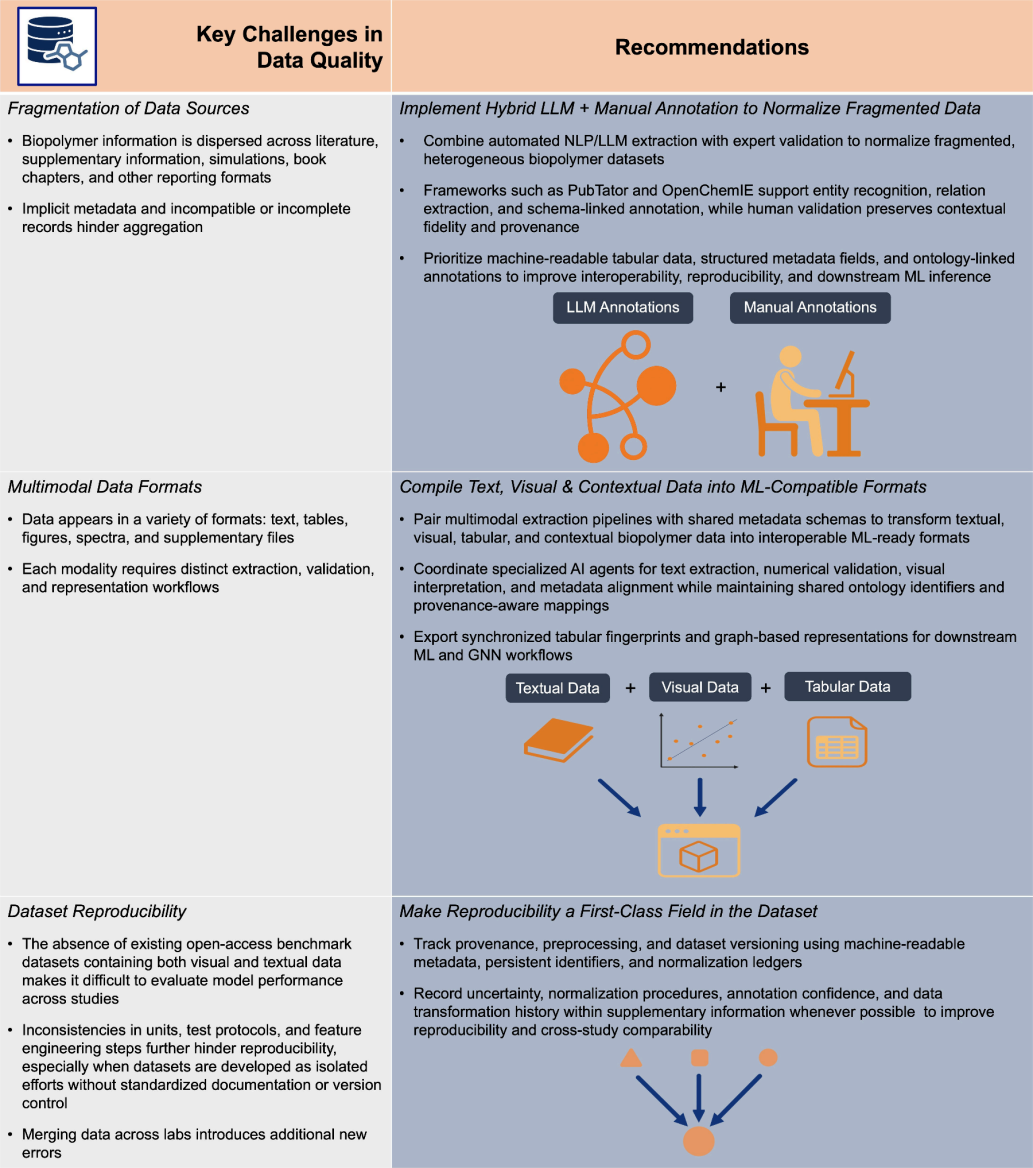
Roadmap for Improving Biopolymer Data Quality^a^

a Three core challenge areas limit the current scalability of biopolymer datasets based on data quality: data source fragmentation, multimodality, and reproducibility (left panel). Hybrid LLM–manual annotation workflows and standardized multimodal data compilation together enable the creation of interoperable, reproducible, and ML-ready biopolymer datasets (right panel).

### 2.1 Key Challenges in Data Quality

#### 2.1.1 Fragmentation of Data Sources

The foremost barrier to curating high-quality biopolymer data sets is fragmentation. Essential descriptors, for example, structure, properties, biosynthesis conditions, processing parameters, and application-relevant measurements, are rarely reported together and instead scattered across studies and supplementary files. While increasing data set size is often emphasized in AI-enabled materials discovery, biopolymer informatics is frequently limited more fundamentally by data quality, reproducibility, provenance, and contextual completeness. Historic and contemporary studies frequently differ in instrumentation and terminology, further complicating alignment.^165,166^ Furthermore, descriptors missing from the literature are often supplemented or substituted with information from available online databases, which may not always be available for the biopolymer composition of interest.^119^ The development and use of biopolymer specific metadata schemas would improve data consistency and compatibility across different resources moving forward. However, new approaches to leverage those schemas need to be developed to curate high quality data sets from the existing literature.

Two clarifications allow us to define the solution space:

- **Aggregation vs normalization**. Aggregation is the act of bringing records together from many sources; whereas normalization is the mathematical and semantic harmonization step, such as value scaling, controlled vocabulary mapping, and schema alignment, that makes those records comparable in ML pipelines. Fragmented data should indeed be aggregated, because aggregation increases sample diversity and statistical power; but must be normalized to be usable.
- **Inconsistency vs incompleteness**. Even with a shared schema, not every study reports every feature. Standardized ontologies and tags (Section 1.1) do not force completeness, but they do ensure compatibility for what is reported, which greatly reduces ambiguity during aggregation and subsequent ML training. Gaps will persist, so normalization must be paired with transparent handling of missingness. “Conflicting metadata” in practice means different phrasings for effectively the same method or condition or undocumented assumptions.^166^ Without controlled terms and explicit fields, curators risk mislabeling or mistakenly merging noncomparable records.^167,168^

In other words, more data is not always better. Similarly, increased metadata complexity does not universally improve ML and AI predictive performance. Rather, clear, standardized methods of data organization, such as embeddings which are directly tailored to a particular application, are essential to improve model training, scaling, and interoperability. In some cases, significant amounts of metadata can be justifiably ignored due to some stages of the polymer lifecycle providing strong features or sensitivity contribution to a model, for example the composition or structural information, whereas the processing or degradation information of that polymer system may not be relevant for the application (and therefore trained model) at hand.

#### 2.1.2 Multimodal Data Formats

Biopolymer data spans textual, visual, and computational outputs. Each modality demands a distinct extraction workflow and yields different levels of structure and confidence.^51^ Text and tables are currently the most amenable to automatic parsing; but while substantial work has focused on information extraction from text and tables,^13,125,159,169^ these methods often rely on evaluation metrics that are highly domain-specific and require expert annotation.

Visual elements contain a wealth of critical quantitative information; however, they often lack machine-readable raw data, and quantitative values must then be digitized, and axis semantics carefully recorded. While recent computer vision methods for processing medical and cellular imaging data are robust and extensive,^170–172^ automated, high-fidelity processing of visual elements in a manuscript – not images – remains limited.^170,173,174^ As a result, it remains an open question how well such approaches can transfer to biopolymer information extraction, where experiments involve numerous interdependent entities and potentially more complex schemas than those considered in existing work.

A second multimodality pitfall is context leakage: biological and environmental conditions are easily implied but not explicitly stated; for example, “under standard conditions” or “as previously described”.^58,169^ LLMs cannot reliably infer such context without schema-anchored fields and manual annotation.^13^ Consequently, end-to-end automation for multimodal data is not yet reliable, despite being ubiquitous for biopolymer data. Human oversight is required to resolve implicit or ambiguous context and to verify data extraction from visual elements.

#### 2.1.3 Data Set Reproducibility

Reproducibility hinges on transparent preprocessing and standardized identifiers. When data sets are merged across laboratories, variability in labeling, unit conversion, and protocol differences can invalidate comparisons if not explicitly encoded.^175,176^ Many published data sets omit descriptions within their Supporting Information and README files: (i) scaling and normalization decisions, (ii) missing data handling and deduplication procedures, and (iii) versioning of both code and data. Without this documentation, downstream users cannot confidently extend the data set.^132^

### 2.2 Recommendations for Standardizing Data Quality

We recommend a three-part strategy to address data quality: (1) hybrid human–LLM curation to aggregate and normalize fragmented sources; (2) multimodal integration that compiles text, visual, and contextual data into ML-compatible formats; and (3) reproducibility infrastructure that records preprocessing, identifiers, and versioning.

#### 2.2.1 Implement Hybrid LLM and Manual Curation to Normalize Fragmented Data

We recommend addressing unknown metadata and experimentally inaccessible variables whenever possible. For both biopolymers and synthetic polymers, scientific limitations such as unknown metadata also contribute to this challenge. In some cases, a variety of the materials, structural, or property data either were never measured or cannot be measured, which are major reasons for their absence in many data sets.^132^ In many materials systems, not just for biopolymers, we acknowledge that the absence of metadata does not necessarily reflect poor reporting practices, but rather genuine experimental or resource limitations in measuring dynamic or heterogeneous material states.^124^ However, these omissions do limit the ability of current libraries to represent the real structural variability that governs downstream processing and measured properties.

While we recommend the normalization of dispersed, heterogeneous literature and data files into standardized records with aligned schemas wherever possible, we recognize that this strategy has constraints and may not be ultimately beneficial for specific applications. We recommend using modern LLM and manual hybrid annotation strategies to mitigate these scientific limitations whenever possible and whenever needed, to take advantage of existing resources. Several recent examples provide insight into practical methods of implementing these recommendations:

- **LLM-assisted retrieval and preannotation**. LLMs rapidly parse large corpora to identify candidate data, such as polymer identities and mechanical properties, while human curators confirm accuracy, resolve ambiguities, and enrich metadata.^51,59,60,159,177^ Frameworks such as PubTator 3.0 already integrate retrieval-augmented generation (RAG), named-entity recognition (NER), and relation extraction (RE) to structure polymer-related information in the first pass of extraction.^178^ Human researchers are then able to confirm and refine the entries. In one example, the community might utilize existing methods designed for structure and properties to expand into application-specific data curation. For instance, OpenChemIE provides a general chemical information extraction framework whose NLP and entity-recognition pipelines may be adapted to identify polymer- and biopolymer-specific structural patterns, processing descriptors, and materials-property relationships.^179,180^ We recommend using NER/RE and OpenChemIE with expanded pattern recognition to identify candidate entities and relations. Where possible, this workflow would map synonyms to controlled vocabulary terms from the schemas introduced in Section 1.1. For example, rather than describing a biopolymer as “industrially compostable” to instead specify the conditions under which the composting capability was measured.
- **Human-in-the-loop normalization**. Curators need to validate entities, resolve ambiguous phrasing, and assign missing explicit nulls and inferred values. Ambiguous claims (“as described previously”) should be preserved as tags with a precision note or with a range of numerical values rather than mistakenly filled or by assuming values.
- **Schema conformance**. Store validated records in a graph or tabular schema that references:
  - Structural encodings (*e.g*., BigSMILES/RDKit objects);
  - Processing fields (metadata library from Section 1.1);
  - Measurement descriptors (test method identifiers, instrument specifics).
- **Automated checks**. Run validators for unit consistency, value ranges such as temperature, pH, time, and required fields per measurement type, and flag outliers for review.
- **Visual digitization loop**. Where raw data are unavailable, route figures to a digitization tool. Pair the extracted arrays with explicit sample IDs and method identifiers.

Based on these recommendations, we propose the following action items:

1. **Guidelines and benchmarks**. Release small “gold-standard” corpora with full-text annotations and clear instructions to calibrate tools and measure progress. Examples of these standard data sets should be provided for several different biopolymer types with a range of size, structural complexity, and application environments. A vignette illustrating specific cases of hybrid manual-automated annotation strategies, as well as examples of machine-readable formatting of data are provided in Scheme 3. This vignette also provides additional specific examples and resources for the proposed extraction of meaningful and structured insights from data using LLMs.
2. **Balance task-specific data utility and signal-to-noise trade-offs**. FAIR and AI-ready data sets require not only accessible data, but scientifically interpretable, provenance-aware, and contextually complete representations that preserve experimental uncertainty and evolving knowledge. However, ‘complete’ representations must be tailored to the intended prediction task rather than maximizing data dimensionality indiscriminately. For example, sequence-derived embeddings may adequately capture trends in protein folding or molecular recognition, if that is the only application of interest, and the rheological, degradation, or processing-dependent material behavior may be safely omitted. However, in another example, mechanical performance in hydrated biopolymer networks may depend more strongly on processing history, cross-link density, and mesoscale organization than sequence or composition alone.
3. **Open curation examples**. Whenever possible, we recommend providing templates for data conversion tables, vocabulary mappings, and normalization logs so different groups produce interoperable outputs.

**Scheme 3:**
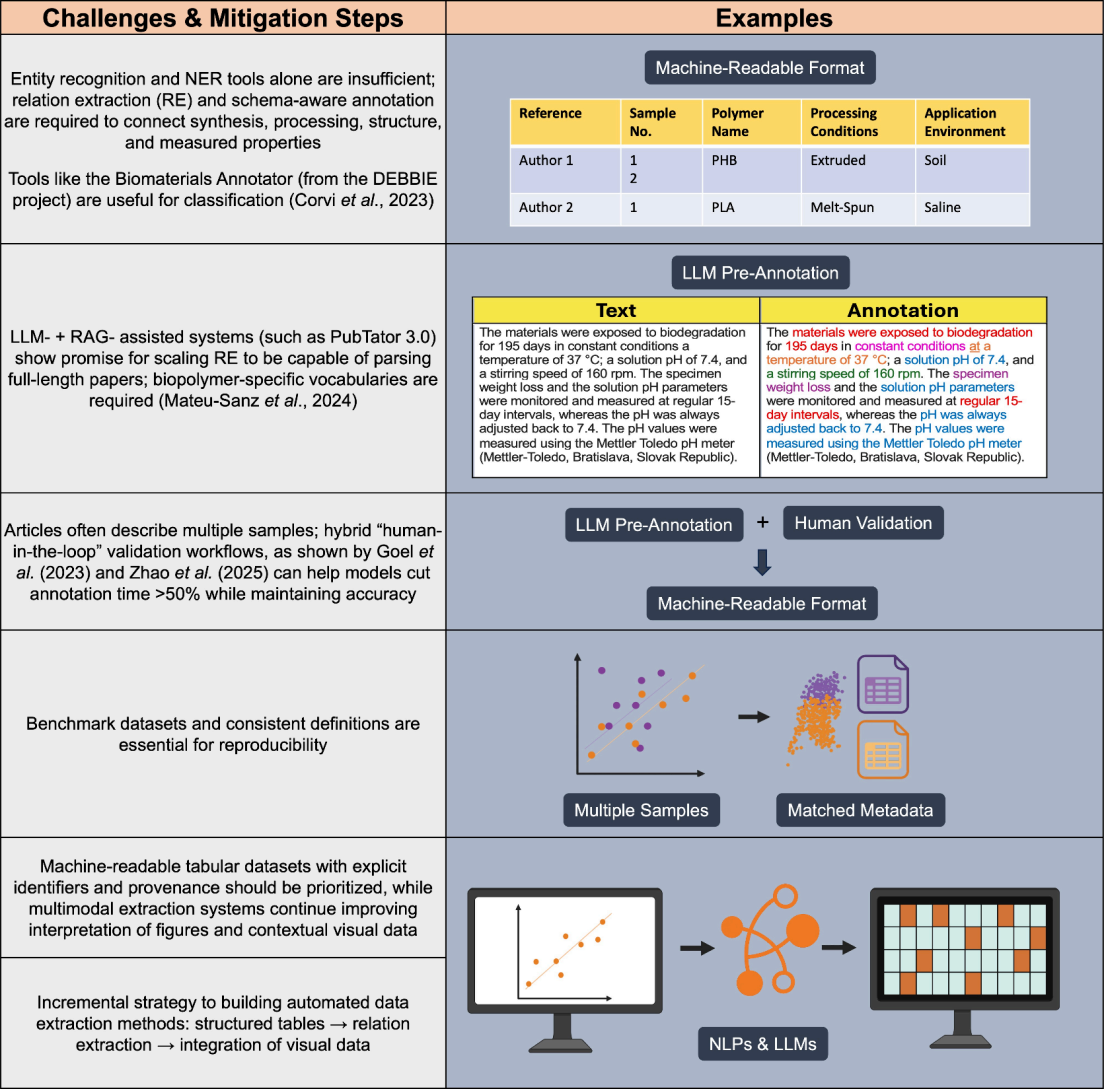
Vignette: Building Structured, Machine-Readable Datasets from Full-Text Biopolymer Literature^a^

a This vignette illustrates how hybrid LLM–human workflows can address the fragmented data problem in biopolymer research using different strategies. The left panel outlines challenges and mitigation strategies, emphasizing the need to move beyond NER to relation extraction (RE), use human-in-the-loop annotation for accuracy, and prioritize machine-readable tables with descriptive identifiers. Benchmark datasets with clear annotation guidelines are also critical for consistency. The right panel provides associated examples: (1) manually converting literature into structured .csv formats, (2) LLM pre-annotation of representative scientific text, (3) hybrid curation of data with domain expert manual annotation, (4) mapping multimodal and multiple samples with matched metadata, and (5) extracting data from visual formats. Together, these steps build toward robust, multimodal datasets that capture synthesis, processing, and property relationships. The sample text for LLM pre-annotation has been adapted from Balagova et al.^181^ and annotations of the sample text are adapted from Kong et al.,^177^ with all required permissions according to creative commons licenses. Citations for each challenge step recommendation are provided directly in the boxes.^13,44,169,182^ .

#### 2.2.2 Compile Text, Visual and Contextual Data into ML-Compatible Formats

To encourage standardized multimodal data sources, we propose curating machine-readable data sets within the data reporting pipeline where every value is paired with identifiers and context, whenever possible for the intended application, enabling direct ingestion by ML pipelines. We recommend the following actions to address multimodality in biopolymer data, with some examples extended in Scheme 3:

- **From text**. Extract, with a combination of manual and LLM/NLP workflows: polymer identity; molecular-weight stats; biosynthetic source, extraction conditions; processing parameters; application testing environment; and measured outputs. Attach method IDs and store with sample-level keys.
- **From visuals**. For example, from a degradation curve, deposit a table with sample_id, method_id, time_unit, time_value, mass_unit, mass_value, plus degradation environment tags. For a Fourier Transform Infrared spectroscopy (FT-IR) peak table or differential scanning calorimetry (DSC) trace, export peak positions/areas or tabulated arrays with instrument and calibration fields.
- **From computation**. Molecular dynamics (MD) and density functional theory (DFT) outputs should be stored with simulation provenance (force field or functional, time step, temperature, box size) mapped to controlled fields so they can be compared with experimental analogs.

Existing resources such as Polymer Genome and LeMaterial^183^ further illustrate this practice for conventional polymers by integrating structure, synthesis, and testing metadata under unified schemas. Building on these frameworks, biopolymer packages should be designed to capture a variety of application-specific metrics by which biopolymers are evaluated. For instance, Polymer Genome represents conventional polymers using chemically informed descriptors, ranging from atomistic and topological to electronic features, and maps them to application-specific metrics through supervised ML models.^30^

This process may be extended to biopolymers. For instance, although LeMaterial does not currently focus on polymer data sets, its standardized approach for linking materials composition, synthesis conditions, and performance measurements through interoperable metadata schemas provides a useful architectural model for future polymer and biopolymer data infrastructures.^183^ Similar architectures could be adapted for biopolymers to integrate hierarchical structure, environmental context, and application-specific performance metrics within FAIR-compliant data ecosystems. A variety of existing cheminformatics packages, such as RDKit, that may be harnessed and expanded to include biopolymer-specific extensions, are also listed in Table 1 as examples of existing high-quality data sets.

**Table 1:** Overview of Existing Cheminformatics Packages^a^

Package Name	Descriptor Information and Utility	Refs and Hyperlinks
Scikit-learn	General ML Toolkit	ref 27
	all-purpose feature extraction, dimensionality reduction, extensive analysis of descriptors from other libraries^27^	https://scikit-learn.org/stable/
RDKit: open-source cheminformatics	Small Molecule Focus	ref 26
	2D structural: molecular weight, LogP, polar surface area, number of rotatable bonds	https://rdkit.org/docs/
	3D structural: molecular shape, conformation-dependent properties, connectivity indices	
	fingerprinting: monomer analysis, Morgan fingerprints, MACCS keys, path-based identifiers^26^	
Matminer	General Materials Science	ref 187
	elemental properties: atomic radii, electronegativity	https://hackingmaterials.lbl.gov/matminer/
	3D structural: crystal structure embedding and crystallinity	
	fingerprinting: chemical formulas, atomic mass, valence, oxidization states^187^	
DeepChem	Deep Learning for Molecules	refs 188 and 189
	graph-based representations: convolutional network modeling of polymer chains	https://deepchem.io
	fingerprinting: extended-connectivity, SMILES, monomer analysis^188,189^	
TensorMol-0.1	Quantum Chemistry ML	ref 190
	graph-based representations: neural network modeling of polymer chains and molecules	https://pubs.rsc.org/en/content/articlelanding/2018/sc/c7sc04934j
	interatomic: atomic environment vectors, electronic structure, quantum chemistry^190^	https://github.com/jparkhill/TensorMol
polymer structure predictor (PSP)	Polymer Informatics	ref 28
	property data: glass transition temperature, melting temperature	https://pubs.acs.org/doi/10.1021/acs.jctc.2c00022
	3D structural: degree of polymerization, branching factor	https://github.com/Ramprasad-Group/PSP
	fingerprinting: monomer analysis, SMILES, InCHI^28^	
biopython	Other Biopolymers (DNA, Proteins, Peptides)	refs 191 and 192
	sequence: nucleotides, codons, amino acids	https://biopython.org
	2D and 3D structural: molecular weight, secondary structure, isoelectric point, hydrophobicity, polarity^191,192^	https://github.com/biopython/biopython

a These packages for encoding biopolymers are available as resources to improve data quality.

Emerging multiagent AI workflows may further facilitate integration of heterogeneous biopolymer data modalities by coordinating specialized agents for text extraction, numerical validation, visual interpretation, and general multimodal retrieval. Recent multiagent frameworks, such as PlotGen suggested by Goswami et al. (2025) demonstrate how orchestrated LLM agents can iteratively refine scientific visualizations using complementary numerical, lexical, and visual feedback loops to improve consistency across raw data tables, generated plots, and textual annotations.^184^ Similarly, hierarchical multimodal retrieval systems such as HM-RAG presented by Liu et al. (2025) illustrate how separate retrieval and reasoning agents may coordinate across structured, graph-based, textual, and visual databases to synthesize context-aware scientific information from heterogeneous sources.^185^ Furthermore, Roy et al. (2026) have also presented an integrated multiagent AI ecosystem for synthetic and biopolymer discovery once the data has been processed, incorporating seven agents into one workflow: an agent for research, characterization, safety, ML modeling, reporting, execution, and synthesis.^186^ Future biopolymer informatics ecosystems should incorporate strategies for harmonizing raw data from different formats as well as automating tasks further downstream in the research pipeline, combining these approaches.

We further suggest the following next steps to demonstrate effective application of these existing resources:

1. Provide two export forms: (i) normalized tabular fingerprints, ready for classical ML; and (ii) graph objects for GNNs where nodes represent materials/process/measurement events and edges encode relationships. Both must reference the same ontology IDs to ensure one-to-one mapping between table columns and graph attributes.
2. Integrate with open cheminformatics libraries, such as an extended RDKit, for featurization and with repositories that already implement FAIR principles.
3. Develop interoperable agentic workflows in which specialized AI agents coordinate extraction, validation, retrieval, and cross-modal alignment of textual, numerical, graphical, and image-based biopolymer data while preserving shared ontology identifiers and provenance-aware metadata mappings.

#### 2.2.3 Make Reproducibility a First-Class Field in the Data Set

To ensure that others can reproduce, audit, and extend the data set and its derived models, we offer suggestions for improving the reproducibility of biopolymer data, specifically as it relates to data quality. The following community standards would improve interoperable, scalable, and trustworthy data sets for training ML models that reflect both structural diversity and environmental context:

- **Provenance and versioning**. Track data sources (DOIs/links), extraction dates, and code versions. Use semantic versioning for data sets, then record changes in a change log.^128,193^

- **Normalization ledger**. Store all unit labels, unit conversions, scaling methods, any data interpolation rules, outlier decisions, and attribution details in machine-readable files. Couple each transformed column to its transformation recipe.
- **Identifiers everywhere**. Use persistent IDs for samples, methods (ASTM/ISO), instruments, organisms/strains, and encodings (for instance, BigSMILES). This allows reliable links between and across contributions and prevents duplicate or mismatched records.
- **Quality flags and uncertainty**. Include fields for measurement uncertainty, digitization confidence, and curator confidence; expose them to downstream ML so models can weight records appropriately.^194–196^

- **Reusability checks**. Provide schema validators and example notebooks that load the data set, reproduce summary plots, and train a baseline model, which demonstrates end-to-end usability.^124,177^

Implementing these recommendations would further improve quality across biopolymer data sets and alignment with FAIR data principles.

## 3 Data Sharing

Effective data sharing is the final link connecting standardized encoding and high-quality data sets to reusable, ML-ready resources. Here, data sharing refers to the publication, distribution, and long-term accessibility of structured, interoperable data sets that include multilevel metadata for structure, properties, processing, and application-specific metrics.^197^ Sharing determines who can access it, under what format, and for how long. Two persistent barriers limit data sharing and progress for implementing FAIR principles to biopolymer data sets: (1) fragmented repositories with poor interoperability, and (2) inconsistent publishing and feature-reporting practices that prevent data reuse across the biopolymer community, which are summarized in Scheme 4. Here, we define *data reuse* as the practice of sharing data sets that were originally collected or created for one purpose to reuse in new workflows. This can involve reusing data for training models, performing meta-analysis, validating results, or conducting comparative studies.

**Scheme 4:**
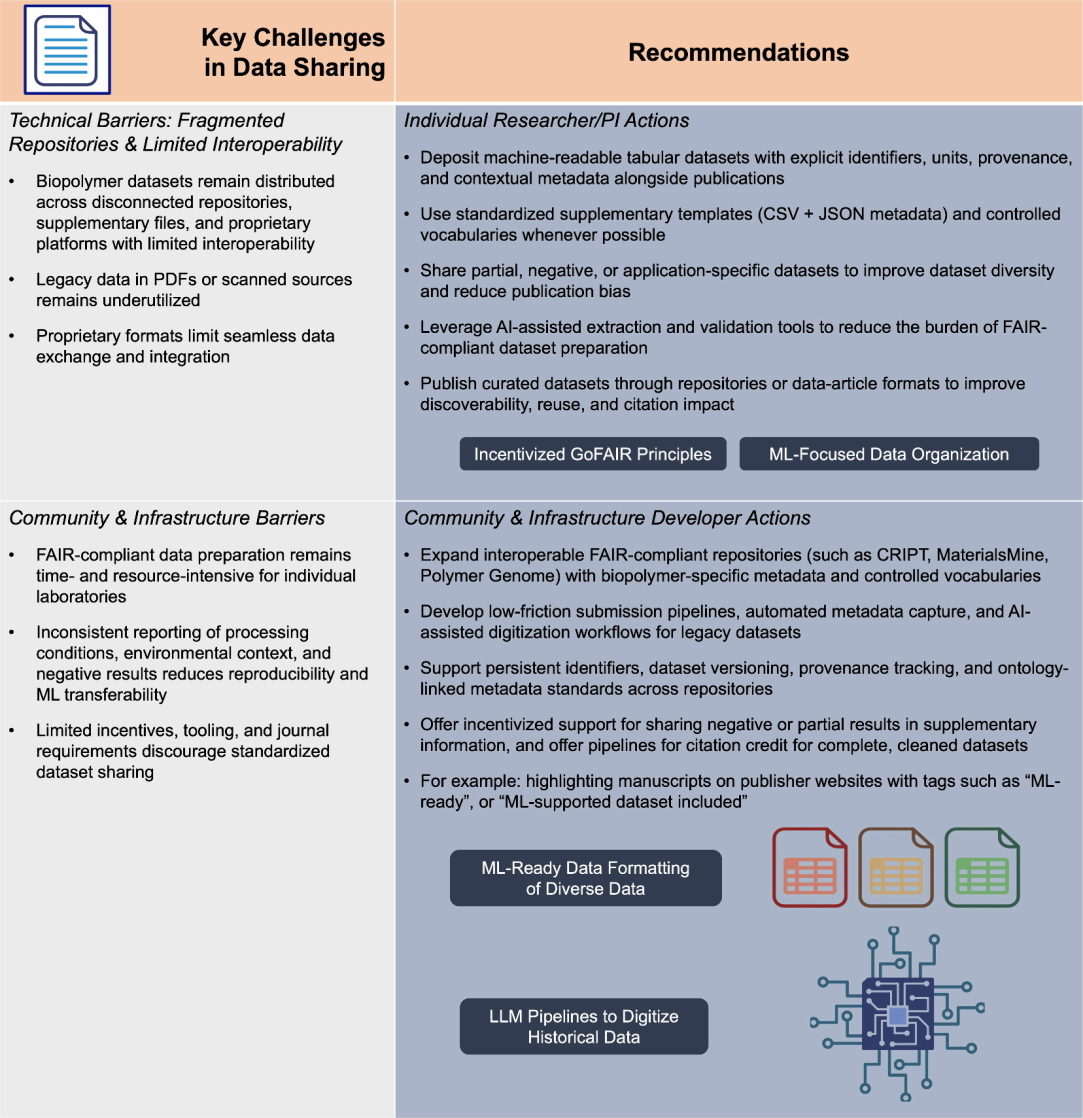
Roadmap for Improving Biopolymer Data Sharing Practices^a^

a The left column outlines the two primary challenges, repository fragmentation and inconsistent reporting, that limit interoperability and reuse of biopolymer datasets. The right column summarizes key recommendations: establishing FAIR-compliant, centralized data hubs and adopting journal-level standards, incentives, and feature-reporting guidelines to ensure long-term accessibility and ML readiness.

### 3.1 Key Challenges in Data Sharing

#### 3.1.1 Technical Barriers: Fragmented Repositories and Limited Interoperability

Current biopolymer data sets are dispersed across independent repositories, journal supplements, and commercial databases, with limited mechanisms for cross-data set integration.^197,198^ Public resources such as PolyInfo, Polymer Genome,^30^ MaterialsMine,^31,147^ and Matmerize^143^ provide extensive infrastructures for synthetic polymers and general materials science, but rarely capture biopolymer application-specific information such as environmental interactions or bioactivity.^15,199^ Specialized data sets for natural or biosynthesized polymers, such as fibroin, cellulose, starch, chitin, or microbial PHAs, also remain sparse or localized to individual laboratories.^7,93^

In contrast, proprietary platforms such as SpringerMaterials,^200^ Total Materia,^201^ and Ansys Granta MI^202^ provide extensive thermodynamic, mechanical, and chemical-resistance data that is compatible with ML. These platforms also include limited biopolymer subsets and specific copolymer and blend formulations.^200–202^ Yet, subscription fees, license restrictions, and export barriers prevent their aggregation into open ML data sets. Even when raw data can be exported, it must be recurated, standardized, and reformatted before integration. Currently the community lacks federated, open-access databases capable of linking structural, environmental, and application-specific metric data for biopolymers under a common schema.

#### 3.1.2 Community and Infrastructure Barriers

Beyond repositories, the lack of consistent publishing standards further limits data reuse. Many data sets appear only as supplementary files in diverse formats without standardized metadata. Although technically machine-readable, these sources require significant manual parsing, which introduces inconsistencies and data loss. From a data sharing perspective, many journals rarely enforce uniform reporting of processing context, application environment, or testing conditions. Thus, identical metrics may appear under different labels or units across studies. Furthermore, many publications do not consistently report negative data or data from failed experiments. This exclusion of data inherently introduces bias in the data set, and in the ML models trained on such data. As observed by Zhu et al.,^197^ inconsistent reporting of sample size, feature size, and experimental context can lead to irreproducible ML outcomes in data. Wang et al.^198^ have also highlighted similar issues broadly in materials science, such as limited specification of data splits, missing metadata, and lack of data set versioning. For biopolymers, inconsistent reporting of feature scope and context translates directly into gaps in shared repositories and, ultimately, lower model reliability and transferability.

Finally, from the standpoint of the researcher, it is resource-intensive to make their data sets FAIR-compliant; and since doing so is often not required or explicitly incentivized by the publishing community, researchers are understandably not inclined to do so. Some journals, such as *Scientific Data* and *Data in Brief*, are specifically focused on publication of data sets. The ability to publish a data set as a separate publication would help to incentivize researchers to make their data sets FAIR-compliant because the data set could be formally cited along with their ‘primary’ publication.

### 3.2 Recommendations for Standardizing Data Sharing Practices

Valuable biopolymer data currently remains in fragmented repositories and inconsistently reported literature, preventing integration across the research community. A unified, interoperable data-sharing ecosystem that links encoding, quality, and access would help close the final gap toward scalable, reproducible ML models to guide biopolymer development. To distinguish near-term researcher actions from longer-term infrastructure needs, we organize data-sharing recommendations across two implementation levels: individual researchers/PIs and infrastructure developers/repository curators. This division of actionable items highlights that FAIR biopolymer data sharing does not require every PI to build infrastructure independently; rather, individual researchers can contribute increasingly structured data sets, while community-level systems can progressively reduce the cost, resources, and investment needed on the part of individual researchers needed for long-term interoperability.

#### 3.2.1 Individual Researcher and PI-Level Actions

At the individual level, researchers can adopt incremental, “low-friction” FAIR practices that remain feasible even under common laboratory resource constraints, including planning data reuse at project initiation, depositing cleaned data sets in available repository-compatible formats, assigning persistent identifiers and licenses, and providing minimal machine-readable metadata describing composition, processing conditions, and measurement context. These steps have been demonstrated within the FAIR materials roadmap proposed by Brinson et al. (2024) to lead to trustworthy, reusable materials data sets and success stories of materials data impact.^203^

Resource limitations at the individual investigator remain a major barrier to FAIR data adoption in both synthetic and biopolymer research. Preparing reusable data sets requires additional time for organization, annotation, metadata generation, and repository deposition, while many laboratories currently lack standardized tools or personnel dedicated to data stewardship.^204^ Rather than expecting immediate comprehensive compliance, we therefore advocate for “minimal viable FAIR” practices that can be implemented incrementally at the laboratory scale, including depositing cleaned tabular data sets, recording uncertainty and missing metadata explicitly, using persistent identifiers and standardized file formats, and linking data sets directly to publications. Even partial implementation of these practices improves discoverability and reuse while helping establish the structured data ecosystems required for future AI-enabled materials science.^205^ Moreover, FAIR data infrastructure itself is expected to reduce long-term researcher burden by decreasing duplicated effort, improving interoperability, and reducing time spent locating or reconstructing prior data sets.^204,205^

Importantly, FAIR-compliant data sharing also provides direct incentives to individual researchers. Analyses of publisher data availability statements have shown that articles linking data sets through repositories receive substantially higher citation impact, with repository-linked data sets associated with a primary journal article seeing total citation increases of up to 25.36% relative to publications without accessible linked data.^206^ These benefits suggest that FAIR biopolymer data practices are not solely based on the data scaling that fuels model ML/AI workflows, but these principles also provide practical mechanisms for increasing data discoverability, more citations, greater recognition, improved credibility and longevity, and expands scientific impact in quantifiable ways. Across multiple scientific domains, structured and interoperable data sets have enabled scaled scientific learning and modern AI/ML workflows that connect sequence, structure, processing history, and function in ways that are difficult to achieve with isolated studies alone. This is true in diverse fields such as biopharmaceutical R&D^207^ and in general computational workflows,^129^ which provide examples of implementing FAIR-compliant data to achieve excellent model performance as well as new insights into the scientific utility of the data sets. As an example within the polymer community, large, annotated polymer materials data sets such as the Dynamic Polymer Annotated Library (DPAL) illustrate how standardized metadata and automated tagging frameworks can transform thousands of disconnected publications into searchable, AI-compatible knowledge resources that identify underexplored chemistries, reveal field-wide trends, and accelerate materials discovery.^133^

To build open, connected infrastructures that support structured formats, standardized metadata fields, and automated data set curation pipelines aligned with the FAIR principles, we recommend the following key actions:

1. **Prioritize raw, tidy data**. Encourage authors to deposit the data underlying plots with columns for sample ID, variable names, and method identifiers. Where raw data cannot be provided, ensure digitized values retain explicit axis semantics and measurement units, and any relevant reference information if sourced from an online database.^78,208^

2. **Establish minimal feature sets**. Community-driven guidelines for experimental characterization data should also define minimum required fields wherever possible and allowable proxies for missing attributes. These should be maintained collaboratively by repository curators and ideally also journal editors to ensure alignment.
3. **Separate facts from assumptions**. Encode explicit measurements in numeric fields; encode context as controlled tags with identifiers. Avoid “silent inference”: if context is truly unknown, record it as unknown.
4. **Use shared schemas**. Align with the structural ontology and the processing and environmental metadata library from Section 1.1 so that text, visuals, and computational outputs share the same identifiers and terms.
5. **Leverage existing frameworks**. Expand proven infrastructures, such as the Polymer Genome, MaterialsMine, and CRIPT, with biopolymer-specific nodes and metadata libraries. This includes fields for biosynthesis conditions, extraction conditions, processing conditions, and performance outcomes. Currently, the protein engineering and modeling community leads the biopolymer community in the data infrastructure that they have developed. Protein informatics demonstrates that standardized data and learned representations can enable predictive ML at scale; however, extending these approaches to broader biopolymers requires incorporation of materials performance information (such as processing, environmental, and hierarchical context) beyond sequence alone. The nonprotein biopolymer community could learn and leverage much from the successful examples set by the protein bioinformatics community. In turn, the emerging protein-based materials community could benefit from greater data set cross-fertilization with the polymer materials informatics community.^209^
6. **Integrate real-time data capture**. Encourage electronic lab notebooks and automated instrument logging to export data directly into standardized templates at the point of collection. Minimize posthoc curation by embedding metadata at the source (biosynthesis to processing to material testing and performance in an application environment).

A coordinated, FAIR-compliant ecosystem at the individual PI level would enable interoperability among diverse repositories and facilitate reproducible, cross-laboratory ML studies for biopolymer discovery and development.

#### 3.2.2 Infrastructure, Repository, and Publisher-Level Actions

At the infrastructure level, repository developers, publishers, and data curators should build systems that reduce the burden on individuals through biopolymer-specific metadata templates, persistent identifiers, standardized submission formats, APIs, benchmark data sets, repository certification metrics, and citable data set records. The benefits of data compliance with FAIR principles also extend to the broader research community, including improved teaching and collaboration practices, an increased community trust in research, easier access to research, improved reproducibility, as well as reducing unneeded experiments that replicate historical data and promoting novelty and innovation in published data sets and the associated publications.^203–205^

To enhance existing publishing strategies toward widespread ML applications, and enhanced compliance with FAIR data requirements, we recommend that newly generated data adhere to the following standardization protocols. These strategies will ensure all future biopolymer data sets are immediately usable and interoperable with ML and other advanced data analysis techniques:

- **Create a centralized biopolymer data portal**. Host through established entities such as the NIST Materials Resource Registry^210^ to ensure long-term stability and governance.
- **Automate ingestion and digitization**. Implement LLM/NLP-based pipelines to extract structured data from legacy literature and scanned documents, converting them into standardized machine-readable formats. These workflows mirror those discussed in Section 1.2 but now feed directly into the centralized repository for permanent archiving.
- **Adopt transparent versioning and licensing**. Require open licenses, persistent identifiers (DOI, ORCID), and full documentation of cleaning and transformation steps. Version control ensures that data sets remain living resources that can evolve without losing provenance. Wang et al.^198^ have previously emphasized version control, open licensing, and full documentation of data processing and cleanup to support reproducibility in broader materials science areas.
- **Standardized submission formats**. Journals should provide structured supplementary data set templates (for example, CSV + JSON metadata) at submission, mirroring repository schemas. Manuscripts could include tags or keywords such as *FAIR-compliant*, *ML-ready*, or *multiproperty data set* to indicate data set completeness. Going beyond recognition at the journal level, publishers should recommend not only a dedicated repository for data but also provide recommendations to data-specific journals or instructions for receiving a citable DOI for their supplement or data set. Some publishers, such as the Royal Society of Chemistry and Elsevier, already do this and provide excellent examples of how this can be effectively implemented to maximize citation credit to authors. This would also provide incentive for the journal as well to maximize visibility.
- **Encourage structured data papers**. Dedicated “data briefs” or companion data set articles, perhaps as a separate article type, allow researchers to publish curated ML-ready data separate from hypothesis-driven manuscripts. This would both incentivize wide-scale data sharing and improve discoverability and credit attribution.
- **Broaden what qualifies as shareable data**. Journals should accept high-quality negative or partial results, clearly labeled and documented, as legitimate data set contributions. These results help balance training data and reduce publication bias. In environmental research, Zhu et al.^197^ have previously proposed best practices such as explicit documentation of sample size, feature ratio, data splitting methods, feature scaling, hyperparameter tuning, and model explainability to ensure data set validity and comparability. Similarly, Wang et al.^198^ stress full source-code and data availability under open licenses and the use of version-controlled repositories for materials science data at large.
- **Digitize historical literature**. Future work with LLM/NLP pipelines can process archived PDFs to populate standardized repositories, closing legacy gaps and improving temporal coverage of biopolymer data.
- **Provide incentives and curation support**. Introduce citation credit for data sets, separate DOIs for supplementary data packages, and integration with open repositories. Examples of available infrastructures for this approach include Zenodo, Harvard Dataverse, Mendeley Data, and Figshare. Publishers could highlight compliant papers with metadata badges or searchable tags. This is already done by several publishing groups, such as the Royal Society of Chemistry, Springer Nature, and the American Chemical Society.

Incremental FAIR adoption at the laboratory level, combined with community-scale repository and metadata infrastructure, would provide a realistic pathway toward AI-ready biopolymer data ecosystems and reducing redundancy in future publications.

## Outlook

As ML becomes increasingly integral to materials research, its application to biopolymers presents distinct challenges that demand domain-specific strategies. The biosynthetic origins, structural irregularity, and environmental sensitivity of biopolymers make it difficult to apply workflows designed for synthetic polymers without adaptation. However, the path forward is increasingly clear: with standardized encoding systems, structured metadata, and hybrid human–LLM approaches for feature extraction, researchers could begin to transform fragmented, unstructured data into coherent, ML-ready resources for the community. The alignment of biopolymer research with FAIR and interoperable data standards would make these data sets not only more discoverable, but also more reusable and comparable across laboratories and disciplines.

The integration of LLMs with cheminformatics and materials informatics platforms will also be essential to unlock insights buried in the unstructured literature and to enable scalable data set assembly. Scientific journals, data set publishers, and researchers all have a shared responsibility to improve transparency by encouraging structured supplementary data and metadata-rich formats that support automation, reproducibility, and reuse. Long-term success will also depend on the development of sustained, interoperable, and readily available infrastructure. This raises a critical question: who pays? Building and maintaining robust data platforms requires more than goodwill; it necessitates continuous funding for curators, software developers, and infrastructure managers. In the biosciences, agencies like the NIH have long supported databases such as the PDB and GenBank as essential community resources. Informatics for the growing field of biopolymer materials requires similar commitments, whether through government funding, industry-academic partnerships, or community-supported models incentivized by citation and usage rewards.

Finally, biopolymer informatics is inherently interdisciplinary. Its progress depends on collaboration among polymer chemists, materials scientists, bioengineers, data scientists, and computational researchers working under shared standards and open communication. By uniting these communities through common frameworks for information encoding, data quality, and data sharing, the field can accelerate discovery and deployment of sustainable, bioderived materials — bridging biology and materials science through an enduring data infrastructure.

Crucially, we also emphasize that biopolymer informatics does not stand alone. This field intersects with synthetic polymer researchers, experimentalists, simulation experts, and the broader materials informatics community. As these groups converge, stronger communication channels and collaborative infrastructure will be vital. Shared standards and cross-disciplinary engagement are not just desirable; they are foundational to accelerating materials discovery with a data infrastructure built for the future.

## Data Availability

All research results and data are presented in this manuscript. No software or code have been included as part of this Perspective.
